# Rainbow-Seq: Combining Cell Lineage Tracing with Single-Cell RNA Sequencing in Preimplantation Embryos

**DOI:** 10.1016/j.isci.2018.08.009

**Published:** 2018-08-16

**Authors:** Fernando H. Biase, Qiuyang Wu, Riccardo Calandrelli, Marcelo Rivas-Astroza, Shuigeng Zhou, Zhen Chen, Sheng Zhong

**Affiliations:** 1 Department of Bioengineering, University of California San Diego, San Diego, CA 92130, USA; 2 Department of Computer Science and Technology, Tongji University, Shanghai 201804, China; 3 School of Computer Science, Fudan University, Shanghai 200433, China; 4 Department of Diabetes Complications and Metabolism, City of Hope, Duarte, CA 91010, USA

**Keywords:** Preimplantation, Cell fate, Single cell, Lineage tracing, Transposon

## Abstract

We developed the Rainbow-seq technology to trace cell division history and reveal single-cell transcriptomes. With distinct fluorescent protein genes as lineage markers, Rainbow-seq enables each single-cell RNA sequencing (RNA-seq) experiment to simultaneously decode the lineage marker genes and read single-cell transcriptomes. We triggered lineage tracking in each blastomere at the 2-cell stage, observed microscopically inequivalent contributions of the progeny to the two embryonic poles at the blastocyst stage, and analyzed every single cell at either 4- or 8-cell stage with deep paired-end sequencing of full-length transcripts. Although lineage difference was not marked unequivocally at a single-gene level, it became clear when the transcriptome was analyzed as a whole. Moreover, several groups of novel transcript isoforms with embedded repeat sequences exhibited lineage difference, suggesting a possible link between DNA demethylation and cell fate decision. Rainbow-seq bridged a critical gap between division history and single-cell RNA-seq assays.

## Highlights

• Rainbow-seq traces cell division history and reveals single-cell transcriptomes
• A single gene does not exhibit unequivocal lineage difference at 4- and 8-cell stages
• Lineage difference becomes clear when the transcriptome is analyzed as a whole
• Novel RNA isoforms with embedded repeat sequences exhibit lineage difference

## Graphical Abstract

**Figure:**
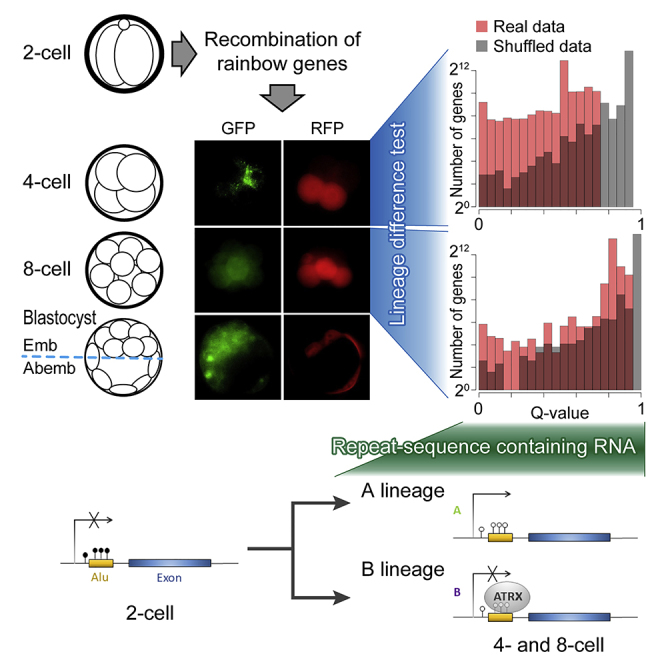

## Introduction

A central question to developmental biology is how cells break molecular and functional symmetry during mitotic divisions. Two models have been proposed (Altschuler and Wu, 2010). In one model, a progenitor cell first divides into a set of homogeneous cells, and then these homogeneous cells exhibit different tendencies toward different routes of differentiation (homogeneous model). In the other model, every division creates a pair of cells with slight differences in their molecular profile, and accumulation of these differences leads to cell fate commitment (non-homogenous model).

**Figure 1:**
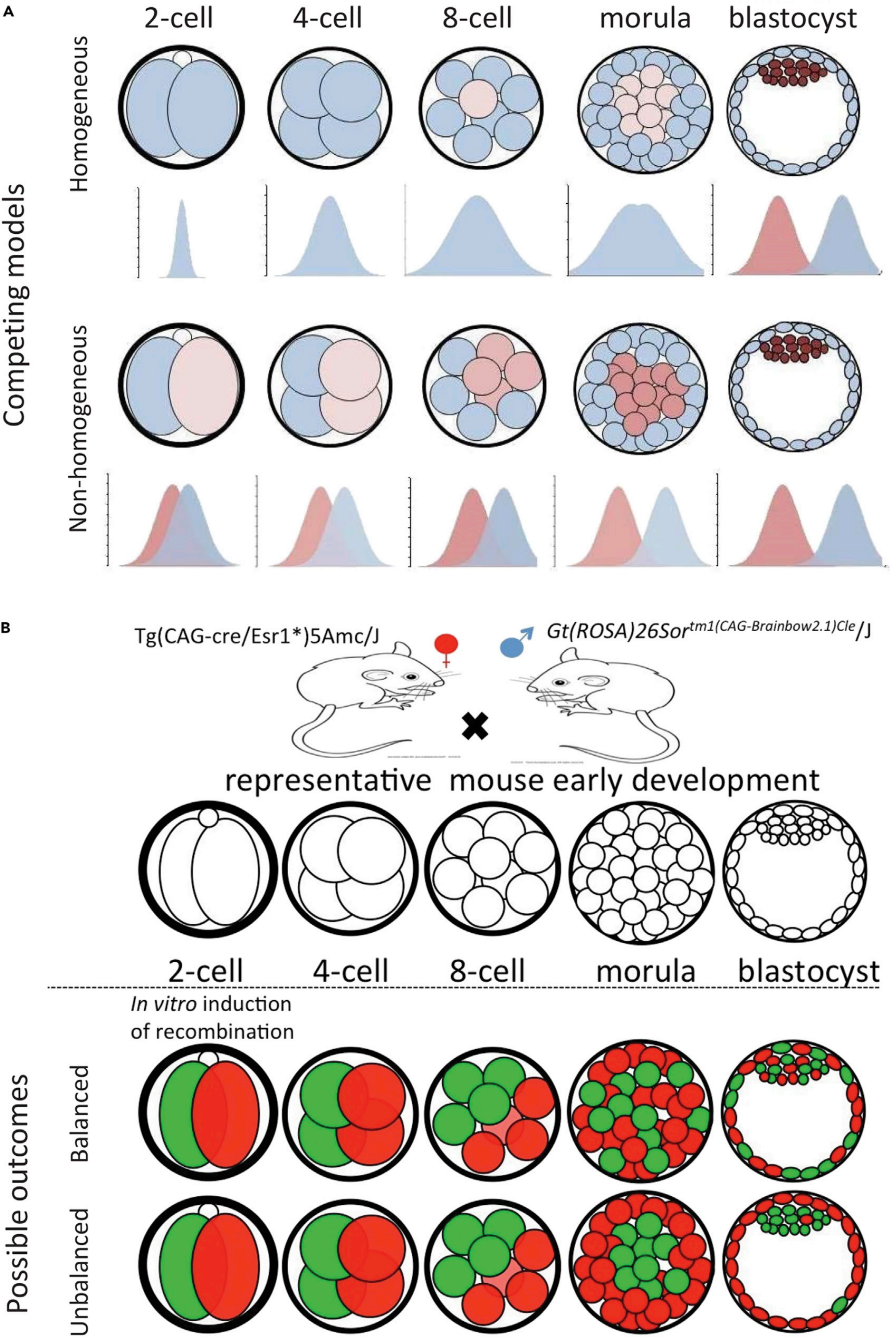
Competing Models and Lineage Tracking Strategy (A) Homogeneous and non-homogeneous hypotheses differ in whether the 2 blastomeres at 2-cell stage have identical tendencies to contribute their descendant cells to inner cell mass (brown) and trophectoderm (blue-gray). (B) Cell lineage tracing was enabled by creation of preimplantation embryos heterozygous for Brainbow sequence and Cre and induction of Cre at 2-cell stage. At blastocyst stage, activated Brainbow genes can exhibit either relatively equal (balanced) or biased (unbalanced) distributions in inner cell mass and trophectoderm. The equal and biased distributions would lend support to the homogeneous and the non-homogeneous hypotheses respectively.

Mammalian preimplantation development from a zygote to a blastocyst consisting of the functionally differentiated inner cell mass (ICM) and trophectoderm (TE) (Wolpert, 2015) is a perfect example of the symmetry-breaking paradigm (Gardner, 2001, Zernicka-Goetz et al., 2009). The first cell fate decision in mammals was believed to be an example of the homogeneous model (Hiiragi et al., 2006). After fertilization, a mouse zygote was thought to undergo two or more rounds of symmetric cell divisions, which create four or more homogeneous embryonic cells (blastomeres) (Leung and Zernicka-Goetz, 2015, Motosugi et al., 2005). These homogeneous cells further divide and through a stochastic process (Wennekamp and Hiiragi, 2012) eventually self-organize into ICM and TE (Wennekamp et al., 2013) (Figure 1A). However, recent work on single-cell transcriptome profiling reported reproducible inter-blastomere gene expression differences in 2- and 4-cell mouse embryos (Goolam et al., 2016, Biase et al., 2014, Burton et al., 2013, Piotrowska-Nitsche et al., 2005, Shi et al., 2015), offering support to the non-homogeneous model (Vogel, 2005, Hiiragi and Solter, 2006).

The critical missing information for using single-cell transcriptome profiling to test the competing models is the history of cell divisions (Goolam et al., 2016, Plachta et al., 2011, Torres-Padilla et al., 2007, Rodriguez-Terrones et al., 2018). We approached the challenge of connecting cell-to-cell transcriptome differences at later embryonic stages to progenitor cells by developing Rainbow-seq, which combines cell division lineage tracing (Tabansky et al., 2013) with single-cell RNA sequencing.

Advances in cell-specific genomic barcodes are revolutionizing cell lineage tracing. Leveraging genome editing tools (McKenna et al., 2016, Kalhor et al., 2017, Perli et al., 2016) (Raj et al., 2018) or combinatorial DNA recombination loci (Polylox) (Pei et al., 2017), researchers were able to insert cell-specific exogenous DNA barcodes to the genomes of progenitor cells. The lineage information is propagated by DNA duplication and can be recovered in the progeny by targeted sequencing of the engineered genomic sequences. We reasoned that combining lineage-specific genomic barcodes and single-cell RNA sequencing (RNA-seq) would reveal single-cell transcriptome together with cell division lineage. To maximize the chances of obtaining expressible genomic barcodes, we resorted to the well-tested Brainbow-2.1 construct that can express one of four fluorescent protein genes (green fluorescent protein [GFP], cyan fluorescent protein [CFP], red fluorescent protein [RFP], yellow fluorescent protein [YFP]) (Livet et al., 2007). These fluorescent protein genes interspersed with recombination sites serve as both genomic barcodes and expressible cell markers. Their expression can be examined microscopically, offering an intermediate step of validation before single-cell RNA-seq analysis.

## Results

### Cell Lineage Tracing Starting with 2-Cell Embryos

**Figure 2:**
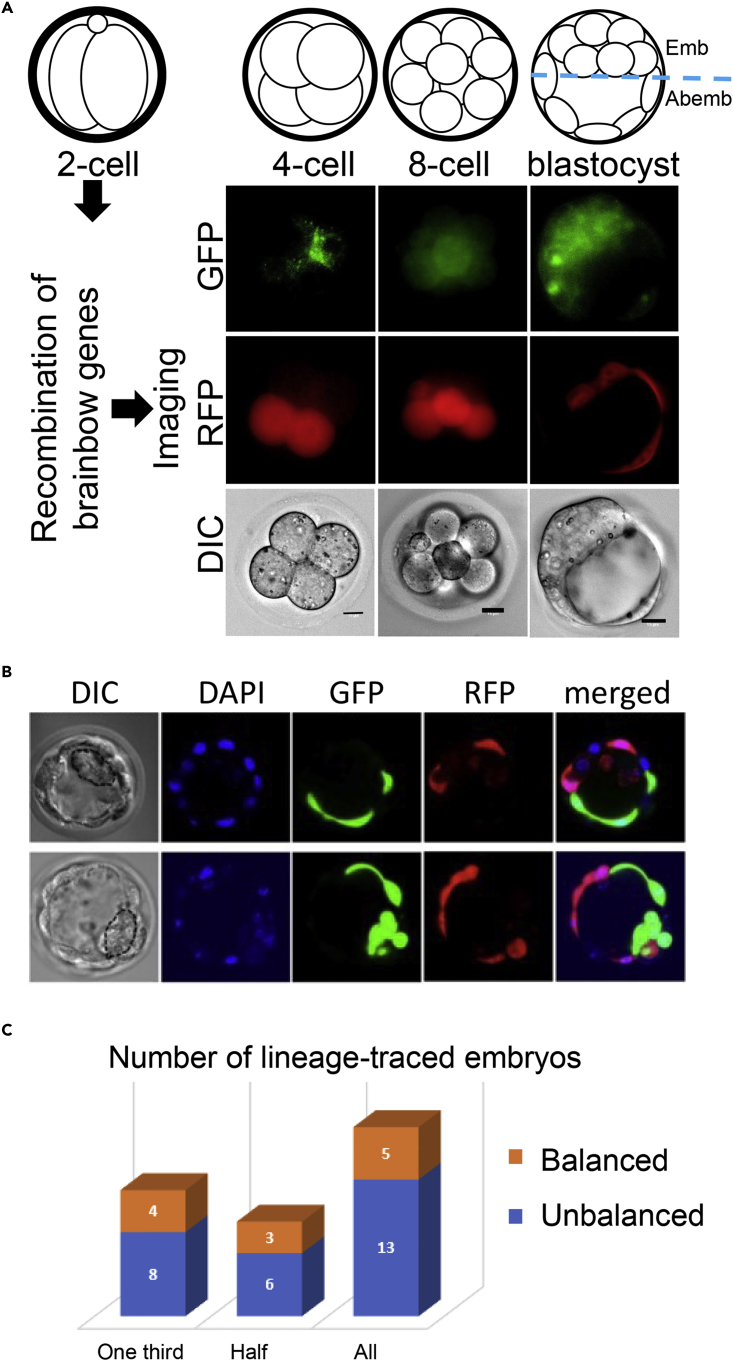
Cell Lineage Tracking in Mouse Preimplantation Embryos (A) Recombination of Brainbow construct was induced in early 2-cell embryos, and images were acquired at 4-cell, 8-cell and blastocyst stages. GFP: GFP, green fluorescent protein; RFP, red fluorescent protein; DIC: differential interference contrast. Scale bar: 15 μm. Images were acquired by a wide-field microscope containing filters for RFP and GFP. (B) Two representative blastocyst-stage embryos imaged with DIC, DAPI, GFP, and RFP channels. (C) Numbers of blastocyst-stage embryos with balanced (orange) and unbalanced (blue) lineage marker expression in the two embryonic poles, with respective counting methods (three lanes). Data from different counting methods are shown in columns, which include the method requiring at least 1/3 cells (one-third) or 1/2 cells (half) expressing either GFP or RFP, and the method of counting all the imaged embryos (all). See also Figure S1.

We generated mouse embryos by crossing a strain that is homozygous for the Brainbow sequence (Cai et al., 2013) with a mouse strain that is homozygous for a tamoxifen-inducible Cre recombinase gene (Figure 1B). Tamoxifen treatment was expected to induce CRE translocation to the nucleus, which in turn could induce recombination on *loxP* sites, and thus lead to activation of one and only one of the four fluorescent protein genes in the Brainbow sequence (Cai et al., 2013). We tested whether these anticipated effects could be achieved by a short application of tamoxifen to early 2-cell embryos (Figure S1A). Upon collection of early 2-cell embryos (42 hours post injection) and a 2-hr 4-hydroxytamoxifen treatment, CRE protein translocated to the nuclei (Figure S1B), and within 1 hr post treatment the nuclear concentration of CRE reduced to the background level (Figure S1B). Embryos that underwent this brief tamoxifen treatment expressed fluorescent proteins at later developmental stages including 4-cell, 8-cell, and blastocyst stages (Figure 2A). Hereafter, we name these early 2-cell-stage hybrid embryos that undergo a 2-hr 4-hydroxytamoxifen treatment as *rainbow embryos*, and the Cre-activated genes as *lineage marker genes*.

### Daughter Cells of Blastomeres at 2-Cell Stage Tend to Contribute Inequivalently to Embryonic and Abembryonic Poles at the Blastocyst Stage

We leveraged the Brainbow embryos to revisit the question whether the descendant cells produced by each blastomere of a 2-cell embryo contribute equivalently to embryonic and abembryonic poles in blastocyst-stage embryos (Hiiragi and Solter, 2004, Plusa et al., 2005, Vogel, 2005). Our experiment was designed as follows. First, a total of N blastocyst-stage rainbow embryos would be imaged by a wide-field fluorescent microscope with filters for GFP, RFP, and DAPI (Figures 2A and 2B). Because we had to devote one of three channels to nuclei staining (DAPI) for cell counting, this experimental design would not be able to detect CFP- and YFP-expressing cells. Next, the imaged embryos with more than one-third of their cells in the embryonic pole expressing a detectable lineage marker (either GFP or RFP) would be used for data analysis. A contingency table would be built for each embryo documenting the numbers of GFP-expressing (GFP+) and non-GFP-expressing (GFP-) cells in the embryonic pole and in the abembryonic pole. An odds ratio (OR) would be calculated for each embryo, and if 1/3 < OR < 3, this embryo would be regarded as having balanced GFP+ cells in the two poles, otherwise the embryo would be regarded as having unbalanced GFP+ cells in the two poles. The same analysis would be carried out for RFP.

We imaged 18 early blastocysts expressing RFP and/or GFP, of which 12 embryos had more than one-third of cells expressing fluorescent protein. Eight of these embryos (66%) exhibited unbalanced GFP+ or RFP+ cells in the two poles (first column, Figure 2C). For sensitivity analyses, we used two alternative counting methods to re-estimate the proportion of embryos with unbalanced distribution of cell lineage between the two poles. First, we required the embryos to be included for analysis to have at least 50% of its cells expressing either GFP or RFP, resulting in a total of 9 embryos, of which 6 embryos (66%) exhibited unbalanced GFP+ or RFP+ cells in the two poles (second column, Figure 2C). Second, we counted the GFP+ cells in the embryonic pole (n1) and GFP+ cells in the abembryonic pole (n2) and regarded any embryo with 1/3 <n1/n2< 3 as having balanced GFP+ cells, and did the same counting for RFP. All 18 embryos were included in this analysis, of which 13 embryos (72%) were unbalanced (third column, Figure 2C). Taken together, approximately two-thirds of rainbow embryos exhibited unbalanced distribution of the two cell lineages in the two embryonic poles. Previous efforts obtained discrepant estimates of such a proportion (Piotrowska et al., 2001, Hiiragi and Solter, 2004, Plusa et al., 2005, Vogel, 2005). Our results obtained from rainbow embryos were better aligned with those suggesting more embryos exhibiting unequal contribution of the two lineages than those exhibiting equal contribution to the embryonic and abembryonic poles. These microscopic analyses confirmed that the recombination strategy introduced lineage-specific expressible marker genes and served as a sanity check for downstream Rainbow-seq analyses.

### Single-Cell RNA Sequencing Analysis of Rainbow Embryos

We induced lineage tracking from 2-cell stage and carried out Rainbow-seq on nine 4-cell embryos and four 8-cell embryos. We lost one blastomere from an 8-cell embryo during the manipulation. We combined the Smart-seq2 full-length RNA-seq protocol (Picelli et al., 2014) with deep paired-end sequencing and produced paired-end 100-nt sequencing data from a total of 67 single blastomeres, including 36 from 4-cell stage and 31 from 8-cell stage (Tables S1 and S2). One 8-cell-stage blastomere did not pass our quality control as it produced fewer than 3 million uniquely mapped sequencing read pairs (Row E13, B7, Table S2). The remaining 66 cells yielded on average 21 million uniquely mapped read pairs (mm10) per cell (Figure S2A). We used single-cell RNA-seq reads mapped to the Brainbow-2.1 sequence to determine which lineage marker gene was expressed and categorized the cells from each embryo into two groups (lineage A and B) based on its expressed marker gene (Lineage column, Tables S1 and S2). Data were insufficient to resolve three 4-cell-stage embryos (12 blastomeres) and four 8-cell-stage blastomeres due to limited sequencing reads mapped onto any lineage marker gene. We deposited all sequencing data into GEO (GSE106287) for public access; however, we limited our analyses to the 50 blastomeres whose lineage can be traced to either one of the sister blastomeres at the 2-cell stage.

The first two principle components explained 8% of sample variations, where the developmental stage was a major source of variation (Figure S2B). With Fragments Per Kilobase of transcript per Million mapped reads (FPKM) >1 as the threshold for calling expressed genes, the 4-cell-stage blastomeres on average expressed 6,852 coding genes per cell. A total of 15,218 coding genes were expressed in at least one 4-cell-stage blastomere. The 8-cell blastomeres on average expressed 6,474 coding genes per cell, with a total of 15,342 coding genes expressed in at least one 8-cell blastomere.

### Transcriptome-wide Expression Difference between Cell Division Lineages

**Figure 3:**
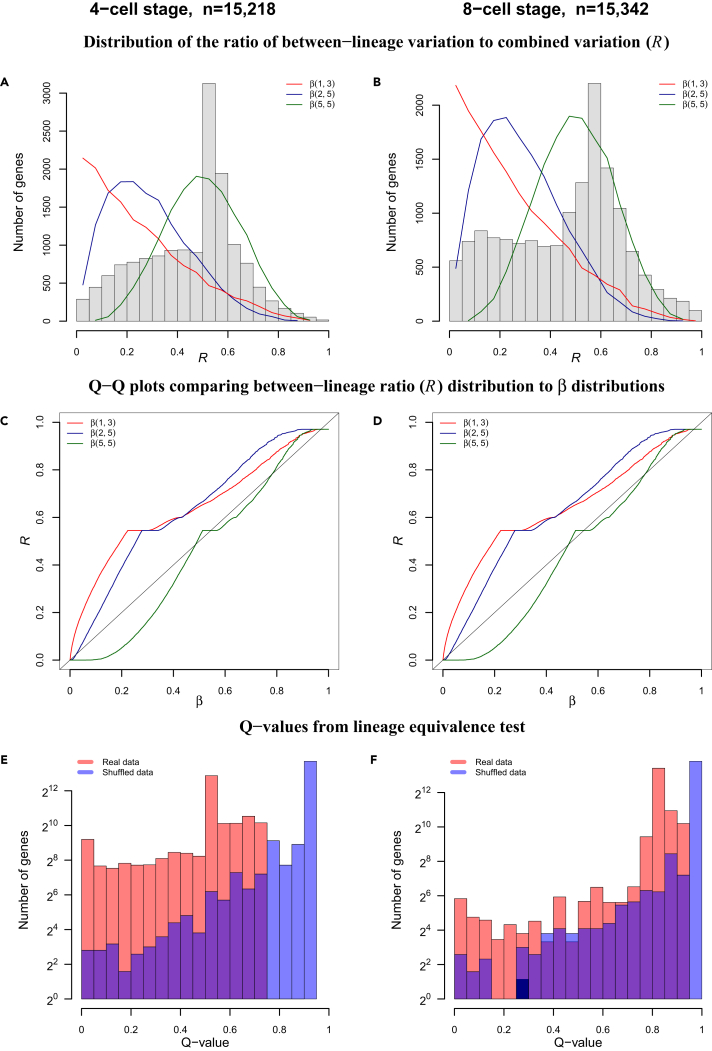
Transcriptome-wide Differences between Two Lineages (A and B) Empirical distributions of random variable R (ratio of between-lineage variation to combined variation) in 4-cell (A) and 8-cell-stage (B) embryos, superimposed with a series of β distributions [red: β(1,3), blue: β(2,2), green: β(5,5)]. (C and D) Q-Q plots of *R* versus a series of β distributions in 4-cell (C) and 8-cell (D) stage embryos, where red, blue, and green represent β(1,3), β(2,2), and β(5,5), respectively. (E and F) Distributions of q-values from lineage equivalence tests based from real data (red) and shuffled data (blue) in 4-cell (E) and 8-cell-stage (F) blastomeres. See also Figure S2 and Tables S1 and S2.

Recognizing that data of individual genes may not provide conclusive evidence to either the homogeneous or the non-homologous model, we analyzed data of the entire transcriptome. We carried out two transcriptome-wide tests. First, we tested whether there is non-zero probability for any subset of genes to exhibit greater between-lineage variation than embryo-to-embryo variation. To this end, we fit a generalized linear model to every gene (*g*) and calculated the sum of squares to account for the between-lineage variation $\left(SS_{lineage\;}^{g}\right)$ and embryo-to-embryo variation $\left(SS_{embryo}^{g}\right)$. For every gene, we calculated the ratio $\left(r^{g}\right)$ of lineage sum of squares to combined sum of squares of embryo and lineage. The empirical distribution of this ratio (denoted as *R*) exhibited greater tail probabilities than a series of three β distributions, β(2,5), β(1,3), and β(5,5), each of which has non-zero probability in any non-singular interval near 1 ([1-δ,1],(δ> 0)) (Figures 3A–3D). The non-zero probability for a subset of genes to exhibit greater lineage variation than embryo variation suggests a transcriptome-wide difference between the two division lineages.

Second, after a gene-by-gene test of the null hypothesis that this gene does not exhibit between-lineage expression differences (ANOVA, Transparent Methods), we obtained the empirical distribution of the q-values for all the genes from such a test (red bars, Figures 3E and 3F). To derive a background distribution, we shuffled the lineage labels on every blastomere and carried out the same test (blue bars, Figures 2E and 2F). The distribution of q-values from real data was skewed toward lower values when compared with the q-values from shuffled data (p value < 2.2 × 10^−16^, Kolmogorov test), reflecting a transcriptome-wide difference between the two cell division lineages.

### Specific Genes with Expression Differences between Cell Division Lineages

**Figure 4:**
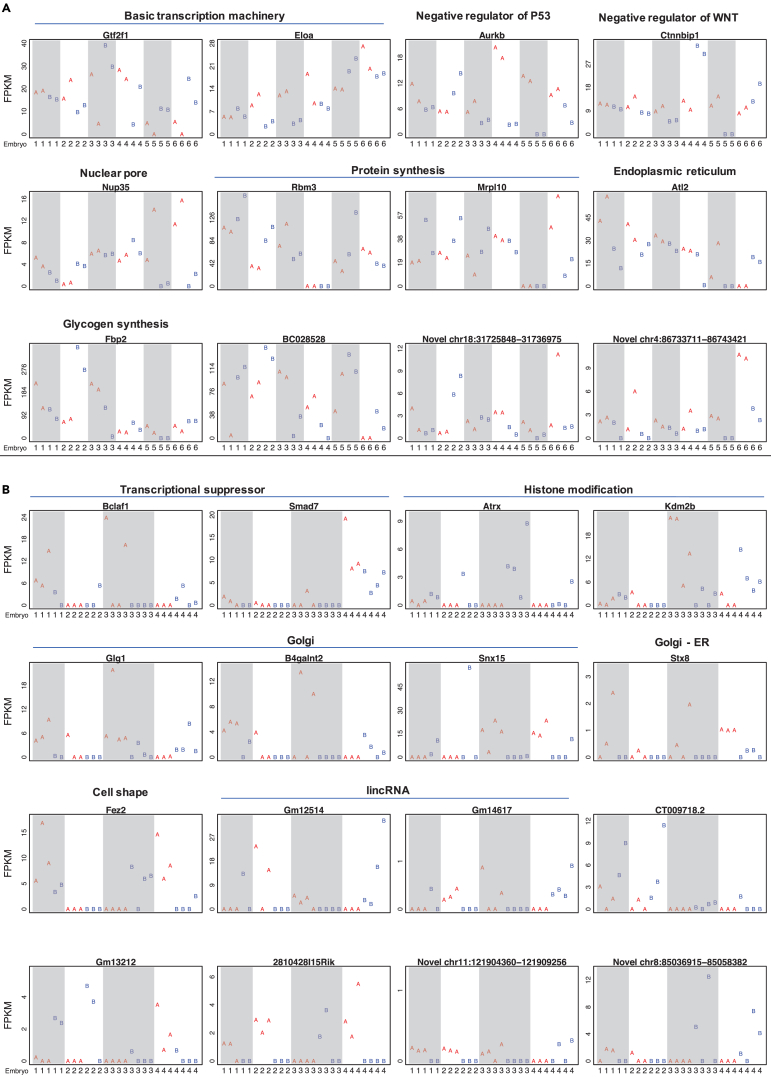
Genes with differential expression between two lineages. Genes with the between-lineage differences at 4- (A) and 8-cell (B) stages. FPKM (y axis) of every blastomere (column) in each embryo (marked by embryo number in columns). Shaded columns delineate different embryos. The blastomeres of the two lineages are marked with A (red) and B (blue).

The genes with the largest between-lineage differences in transcript abundance at 4-cell stage were involved in the basic transcription machinery including *Gtf2f1* (General Transcription Factor IIF Subunit 1, TFIIF) and *Eloa* (RNA Polymerase II Transcription Factor SIII Subunit A1, SIII), negative regulations of P53 activity (*Aurkb*) and WNT signaling (*Ctnnbip1*, Inhibitor of Beta-Catenin and Tcf4), nuclear pore complex (*Nup35*), protein synthesis in cytoplasm (*Rbm3*) and mitochondrion (*Mrpl10*), endoplasmic reticulum (ER) biogenesis (*Atl2*), and glycogen synthesis (*Fbp2*) (Figure 4A). The genes with the largest between-lineage variations at 8-cell stage were transcriptional suppressors *Bclaf1* and *Smad7*, the chromatin remodeler *Atrx* involved in deposition of H3K9me3, histone demethylase *Kdm2b* related to removal of active histone marks including H3K4me3, vesicle trafficking and secretory pathways including Golgi membrane proteins *Glg1* and *B4galnt2*, *trans*-Golgi network, *Snx15* and Golgi-to-ER transport *Stx8* (vesicle fusion, Golgi-to-ER transport), cell shape modulator *Fez2*, and the lincRNA genes *Gm12514* and *GM14617* (Figure 4B). Interestingly, suppression of H3K4 demethylase *Kdm2b* in mouse oocytes resulted in impaired blastocyst formation (see extended Figure 6E in Liu et al. (2016)). Even though the above-mentioned genes appeared to support the non-homologous model, the absolute expression difference is small (Figure 4) and statistical significance is obscure due to limited sample size and multiple comparisons. At this point, it became clear that the statistical tests based on the entire transcriptome (Figure 3) served to better evaluate the two competing models than any possible test based on any individual gene.

### Transposon-Related Novel Transcript Isoforms

**Figure 5:**
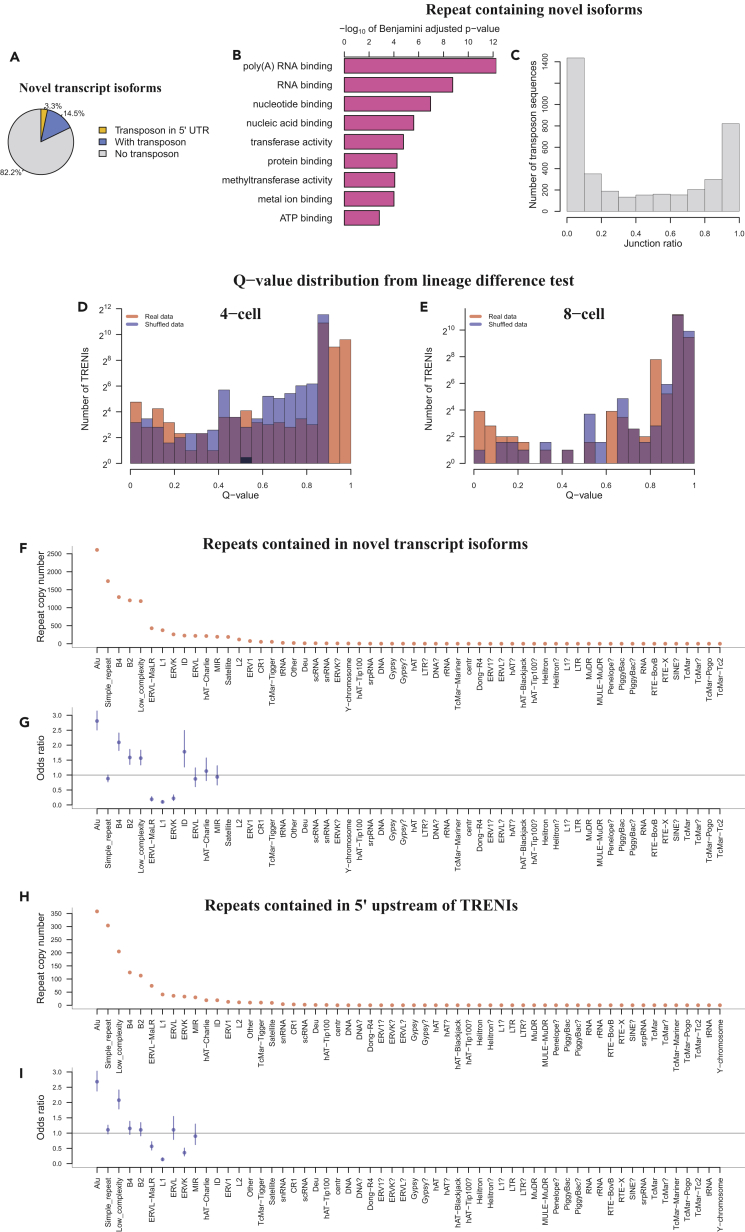
Transposon Related Novel Isoforms (TRENIs) (A) Pie chart of novel transcript isoforms, including 16,739 that do not contain any repeat sequence (gray) and 3,064 and 673 isoforms with transposons located downstream to start codons (blue) and in 5′ UTRs (yellow). (B) Enriched GO molecular functions in TRENIs, ranked by multiple hypothesis testing adjusted p values. (C) Histogram of junction ratios for all TRENI-containing transposons. (D and E) Histogram of TRENIs' q-values derived from tests of equivalent expression between lineages at 4- (D) and 8-cell (E) stages, based from real data (red) and shuffled data (blue). (F) Copy number of repeats contained in TRENIs, categorized by transposon family (columns). (G) Odds ratios between TRENIs and each repeat family (columns). Vertical bars: 95% confidence intervals. Odds ratio was not calculated for those repeat families when the TRENIs associated with a repeat family were fewer than 20. (H) Copy number of repeats contained in TRENIs' 5 UTRs, categorized by transposon family (columns). (I) Odds ratios between 5′UTR-TRENIs and each repeat family (columns). Vertical bars: 95% confidence intervals. Odds ratio was not calculated for those repeat families when the 5′UTR-TRENIs associated with a repeat family were fewer than 20. See also Figures S3–S6.

We tested whether any novel transcript isoforms were specifically expressed in early-stage preimplantation embryos. To this end, we combined all the Rainbow-seq datasets to obtain over 2.1 billion paired-end reads, of which over 1.6 billion read pairs could be uniquely mapped to the genome (mm10). Using Cufflinks (v2.2.1 with g parameter), we recorded a total of 20,371 novel transcript isoforms, corresponding to 7,594 annotated genes. Among these novel isoforms, 3,632 (17.8% of all novel isoforms) contained transposon sequences according to Repeatmasker database (February 2015 release) (Figure 5A), which will be referred to as Transposon Related Novel Isoforms (TRENI). These 3,632 TRENIs corresponded to 1,701 Refseq genes, in which “poly(A) RNA binding,”“RNA binding,” “nucleotide binding,” and “nucleic acid binding” were enriched (Benjamini adjusted p value < 1.1 × 10^−7^; Gene Ontology functions [DAVID v6.8]) (Figure 5B), suggesting a recurring theme in RNA binding and processing.

We asked whether the incorporation of these transposons in TRENIs were supported by junction reads spanning across the transposon and the nearest known Refseq exons. To this end, we computed the ratio of junction reads and the total number of reads aligned to each transposon, hereafter denoted as junction ratio (JR, Transparent Methods). We identified 3,902 transposons contained in TRENIs, of which 2,817 (72.19%) exhibited a JR of 0.9 or above (Figure 5C).

To test whether TRENIs exhibit between-lineage expression difference, we derived a q-value for each of the 3,632 TRENIs by ANOVA (Transparent Methods). For comparison, we shuffled the lineage labels on all blastomeres and derived q-values again (background q-values). The distribution of real data q-values differed with that of background q-values (p value < 2.2 × 10^−16^, Kolmogorov test) (Figures 5D, 5E, and S3). More importantly, a total of 37 TRINEs exhibited q-values less than 0.01, whereas none of the background q-values were less than the same threshold (Figure S3). Finally, all the repeats expressed in 4- and 8-cell embryos, regardless of whether they were contained within TRINEs, did not exhibit statistically discernable between-lineage differences (the distribution of q-values derived from real data was not different from that of shuffled data) (Figure S4).

### Murine Alu Makes the Largest Contribution to Novel Transcript Isoforms

We asked whether different repeat families contributed equally to TRENIs. In the total of 58 repeat families (RepeatMasker) 18 families had 20 or more genomic copies included in TRENIs (Figure 5F). To account for genome-wide copy numbers of each repeat family, we calculated the OR for each repeat family to be contained in TRENIs. When repeat families were ranked by this OR, murine Alu, B4, ID, and B2 were most enriched in TRENIs (Figure 5G). We asked whether the association between any repeat family and TRENIs would became more pronounced when only high-confidence transposon-containing TRENIs were included in the analysis. To this end, we repeated the above analysis using only the 2,817 transposons with JR ≥ 0.9, which resulted in similar ORs with Alu being the most enriched repeat family (Figure S5).

Next, we asked whether different repeat families contributed equally to TRENIs' 5′ UTR. There were a total of 673 (18.53%) TRENIs in which the transposons were inserted in the 5′ upstream to previously annotated start codons (5′ UTR-TRENIs) (Figure 5H), among which 418 contained murine Alu, simple repeat, low complexity, and B4 and B2 repeats in 5′ UTRs, where murine Alu alone accounted for more than three hundred 5′ UTRs (Figure 4H). We calculated the OR to quantify the association of transposons in each transposon family to 5′UTR-TRENIs. Murine Alu exhibited the largest enrichment to 5′ UTRs in these novel transcript isoforms (OR = 3.03, p value = 0, chi-square test) (Figure 4H). In contrast, the other repeat families exhibited either moderate enrichment (1 < OR <1.5) or depletion (OR <1) in 5′ UTR-TRENIs (Figure 5I). Finally, 9 and 26 5′ UTR-TRENIs exhibited expression differences between the two division lineages at 4- and 8-cell stages, respectively, including novel transcript isoforms of *Gata4* (Figure S6A) and *Khdc1a* (Figure S6B).

## Discussion

### Lack-of-Continuity Dilemma

Not a single gene has been reported to exhibit continuous between-lineage expression difference starting from the two blastomeres at 2-cell stage until the formation of ICM and TE at blastocyst stage. This is not completely unexpected considering that comprehensive transcriptome analyses revealed highly dynamic gene transcription in early-stage mouse preimplantation embryos (Kidder and Pedersen, 1982, Xie et al., 2010), likely attributable to combined effects of cleavage-related split of RNA pool, RNA degradation, and zygotic genome activation (Biase et al., 2014). The lack of identified single gene(s) with continuous expression divergence posed a dilemma to the non-homologous hypothesis, in that if the two blastomeres at 2-cell stage are poised for cell fate decisions, what could to be the molecular means to record and later exhibit such a difference? Hereafter we call this dilemma the lack-of-continuity dilemma.

### Reconciliations to Lack-of-Continuity Dilemma

The dilemma was rooted in the non-homogenous model and may be resolved in several ways. First, the cell fate decision is initiated after the first cleavage. This idea essentially refutes the non-homogeneous model for the first cleavage. Second, the lineage difference is passed from one gene activated at an earlier stage, for example, 2-cell stage, onto another gene activated at a later stage. Given that the transcription rate and mRNA half-life are both an order of magnitude smaller than the time of a cleavage, this model is within the boundaries of biophysics principles. Third, the lineage difference is recorded in the order of expression levels of a pair or list of genes, and this order is preserved throughout preimplantation development. For example, at 2-cell stage the expression level order is A > B in one blastomere and B > A in the other blastomere, and such an order is preserved in the ICM and TE. The third explanation was supported by single-cell RNA-seq data (Biase et al., 2014). Taken together, there are plausible means to explain the dilemma while withholding the homologous model.

### The Rainbow-seq Approach

Rainbow-seq addresses the lack-of-continuity dilemma from another perspective. The main idea is to combine cell division tracing with single-cell RNA-seq. A genetic cell-lineage tracer was activated at 2-cell stage and analyzed at later stages. This method has two advantages. First, activation of distinct tracer genes in each blastomere's genome was achieved by a relatively simple procedure. Second, through a genetic tracer embedded in the genome, DNA duplication ensured inheritance of the tracer during cell divisions. We used Rainbow-seq to test the consequential events of two cell division lineages created by the first cleavage.

### The Lineage Difference Lies in the Collective Behavior of Many Genes

When each gene is individually analyzed, even the most significant genes only exhibited small expression differences between the lineages (Figure 4). However, the difference of the two lineages became clear when the entire transcriptome was analyzed as a whole. In other words, the collective distribution of transcriptome exhibited a significant difference in the two cell division lineages (Figure 3). This observation has important implications. On the one hand, it repeated once again the negative results of previous studies that not a single gene exhibited clear difference between the two division lineages created by the first cleavage. On the other hand, the two division lineages in fact exhibit pronounced difference, which lies in the integral expression of many genes. To translate this difficult concept, we would like to make an analogy that “the individual seed beads all look similar, but two bead-woven crafts can exhibit very different patterns.”

Recent studies have reported inter-blastomere expression difference of several specific genes at the 4-cell stage or later stages (Biase et al., 2014, Burton et al., 2013, Piotrowska-Nitsche et al., 2005). This study pushed the initiation time point of between-blastomere difference to the 2-cell stage. The top-ranked genes with between-lineage differences appeared to emphasize a theme of negative regulation, including inhibitors of the WNT pathway *Ctnnbip1* (*Inhibitor of Beta-Catenin and Tcf4*) (Tago et al., 2000) and *Smad7* (Han et al., 2006), *aurora kinase B* that negatively regulates P53's transcriptional activity by promoting its degradation (Gully et al., 2012), chromatin remodeler *Atrx* that promotes repressive histone mark H3K9me3 and formation of heterochromatin (Sadic et al., 2015), histone demethylase *Kdm2b* that removes active histone mark H3K4me3 (Tsukada et al., 2006), and glycosyltransferase *B4galnt2* involved in negative regulation of cell-cell adhesion (Kawamura et al., 2005) (Figure 4). Considering that negative feedback as exemplified in Delta-Notch signaling is perhaps the best known molecular mechanism for neighboring cells to take on divergent functions (Artavanis-Tsakonas et al., 1999), the emergence of negative regulators from Rainbow-seq analysis may allude to cell-cell communication between the first two cell lineages. The other emerged theme of vesicle trafficking and secretion as exemplified by *Atl2*, *Glg1*, *B4galnt2*, *Snx15,* and *Stx8* is consistent with the idea of cell-cell communication at very early embryonic stages (Calarco-Gillam, 1986) (Sozen et al., 2014). Finally, *Fbp2*'s divergent presence in the two lineages is reminiscent of a sperm-related embryo patterning hypothesis (Zernicka-Goetz, 2002, Takaoka and Hamada, 2014), considering the presence of *Fbp2* mRNA in mouse sperm (Schuster et al., 2016) and interactions of FBP2 and ALDOA, a sperm-head protein essential for sperm-egg interaction (Petit et al., 2013).

### Contribution of Transposon Related Novel RNA Isoforms (TRENI) and DNA Demethylation to Lineage Difference

**Figure 6:**
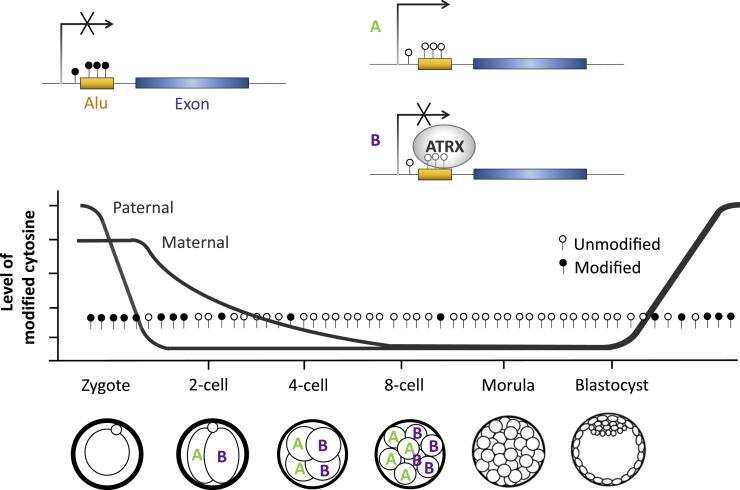
A Model of Repeat Sequence-Related Lineage Difference Decrease of modified cytosine (filled circles) allows repeat sequence (yellow) to be expressed (A). *Atrx* is expressed in one lineage where ATRX preferentially attaches to hypomethylated (empty circles) repeats and suppresses their expression (B).

A group of novel transcript isoforms of known genes that contained repeat sequences, called TRENIs, exhibited lineage difference, especially at the 8-cell stage (Figure 5D). The lineage difference is more pronounced in each group of TRENIs (Figures 5D and 5E) than an individual TRENI (Figure S6), reinforcing the idea that lineage difference at such early stages of development is marked by the cumulative behavior of many genes. At 8-cell stage, chromatin remodeler *Atrx* that responds to DNA demethylation and suppresses the expression of hypomethylated repeat sequence in preimplantation embryos (He et al., 2015) exhibited between-lineage expression difference (Figure 4B). These data suggest a model where DNA demethylation on repeat sequence is asymmetrically compensated by ATRX in the two cell lineages, leading to differential expression of transposon-containing transcripts (Figure 6). Murine Alu repeats were most enriched in TRENIs, and the murine Alu family was the only repeat family with significant enrichment at the 5′UTR of TRENIs. These expression data may offer an explanation to the selective retention of Alu repeats to upstream and intronic regions of known genes (Tsirigos and Rigoutsos, 2009), where the selectively retained Alu repeats are part of rare transcript isoforms, which are specifically expressed in preimplantation embryos. Furthermore, asymmetric suppression of these repeats leads to asymmetric expression of genes required for the first cell fate decision, which is the functional force for selective retention. Consistent with this idea, the same group of genes involved in “RNA binding” were enriched in TRENIs and were reported by an independent analysis as the neighboring genes to selectively retained Alu repeats (Tsirigos and Rigoutsos, 2009). However, the other transcripts of repeat sequences that were expressed in preimplantation embryos (Rodriguez-Terrones and Torres-Padilla, 2018) but not contained within TRENIs did not exhibit statistically discernable lineage differences (Figure S4).

### Limitations of the Study

#### Tradeoff between the Number of Embryos and Sequencing Depth

Our experimental design was determined by our analysis goals. The goals of identifying potentially very small inter-blastomere differences and novel transcript isoforms required deep paired-end sequencing of full-length transcriptome in single cells. Most single cells in this study produced 20 to 30 million uniquely mapped 100-nt read pairs, which are 40–60 million uniquely mapped 100 nt reads per cell. Sequencing cost remains a limitation at this sequencing depth. If future applications do not care about transposon-produced transcripts or novel RNA isoforms, further experiments can significantly reduce the sequencing depth, use single-end sequencing, and even replace with other protocols that do not sequence full-length transcripts. As a tradeoff, more single cells can be sequenced.

## Methods

All methods can be found in the accompanying Transparent Methods supplemental file.

## Data and Software Availability

The GEO accession number for the data reported in this paper is GEO: GSE106287.

## Supplemental Information

Document S1. Transparent Methods, Figures S1–S6, and Tables S1 and S2
