# Supplementary material for: Rainbow-Seq: Combining Cell Lineage Tracing with Single-Cell RNA Sequencing in Preimplantation Embryos
Source: iScience. 2018 Aug 16;7:16–29. doi: 10.1016/j.isci.2018.08.009 (PMC6135740; doi:10.1016/j.isci.2018.08.009)
Supplement: Document S1. Transparent Methods, Figures S1–S6, and Tables S1 and S2 [file mmc1.pdf]

ISCI, Volume 7

Supplemental Information

Rainbow-Seq: Combining Cell Lineage Tracing with Single-Cell RNA Sequencing in Preimplantation Embryos

Fernando H. Biase, Qiuyang Wu, Riccardo Calandrelli, Marcelo Rivas-Astroza, Shuigeng Zhou, Zhen Chen, and Sheng Zhong

Transparent Methods

Generation of rainbow mouse embryos

All the procedures for animal handling and breeding were approved by Institutional Animal Care and Use Committees of University of California San Diego. For the generation of rainbow embryos, we crossed two transgenic strains obtained from Jackson Laboratory. The strain STOCK Gt(ROSA)26Sortm1(CAG-Brainbow2.1)Cle/J (stock number 013731, RRID:SCR_004894) is homozygous for one copy of the brainbow construct v2.1, and the strain STOCK Tg(CAG-cre/Esr1*)5Amc/J (IMSR Cat# JAX:004453, RRID:IMSR_JAX:004453) is homozygous with one copy of the gene coding CRE protein. We crossed mice from each of the strains (1:1) to generate hybrid embryos containing one copy of the brainbow construct v2.1 and one copy of the Cre gene.

Adult female mice were estrous-synchronized and super-ovulated by two consecutive intra-peritoneal injections of hormones (to 2.5 IU of hCG and 5IU of PMSG, PG600, Intervet). The injections were administered 48 hours apart, and following the second injection, females and males were mated (1:1). Two-cell embryos were collected 46 hours post second hormonal injection (hpi) and incubated (37°C, 5%CO_2, 100% humidity) for two hours with 4-Hydroxytamoxifen (100µm, Sigma) in KSOM media (Millipore).

Embryo imaging

Tamoxifen-treated embryos were cultured *in vitro* (37°C, 5%CO_2, 100% humidity) until reaching 4-cell, 8-cell or blastocyst stages (~ 54, 70 and 90hpi, respectively), briefly treated with NucBlue^{TM} Live Cell Stain (Invitrogen) in KSOM media and taken to wild-field inverted fluorescent microscope (Olympus). Embryos were imaged in KSOM media, with filter cubes for DAPI (Ex: 350/50x, Em: 460/50m), GFP (Ex: 480/40x, Em: 535/50m), RFP (Ex: 545/25x, Em: 605/70m) and differential interference contrast, and a 40x dry objective (NA=0.95, WD=0.18mm). Images were captured with an ORCA R2 monochromatic CCD camera (Hamammatsu) at 2µm spacing intervals in the z-axis, and rendered in MetaMorph software (Molecular Devices). Images were further evaluated in ImageJ software (Schneider et al., 2012).

Immunocytochemistry assay

Tow-cell embryos were fixed in methanol at −20°C overnight, washed in PBS (supplemented with 0.1% BSA), and incubated for 5 min in permeabilization solution (PBS, 0.1% BSA, 0.1%

Triton X-100). Embryos were then incubated for 1 h in blocking solution (PBS, 0.1% BSA, 10% bovine fetal serum), following incubation with Monoclonal Anti-Cre antibody (Sigma-Aldrich Cat# C7988, RRID:AB_439697, 1:100 dilution in blocking solution) for 2 h at room temperature. After three PBS (0.1% BSA) washes, indirect detection was possible by immersing the embryos in blocking solution with Goat Anti-Mouse IgG1 (γ1) Antibody, Alexa Fluor 594 Conjugated (Innovative Research Cat# 322588, RRID:AB_1502303, 1:500 dilution). Following three washes with PBS (0.1% BSA), embryos were immersed in blocking solution containing Mouse anti-Tubulin-Alexa-488 (Innovative Research Cat# 322588, RRID:AB_1502303, 1:25dilution) for 1 h. Embryos were washed in PBS (0.1% BSA) supplemented with NucBlue Fixed Cell Stain ReadyProbes (Invitrogen), and preserved with Prolong Gold (Invitrogen). Embryos were scanned with a Fluoview FV1000 confocal microscope (Olympus), with the aid of a 40× oil objective.

Assessment of color-coded distribution of cells in blastocysts

Blastocyst images were visually inspected by one investigator. Cells were identified with the by their nucleus staining with DAPI. The cells were classified according to two criteria: First, cells were assigned to the embryonic (em) or abembryonic (abe) pole of the embryo. Second, the cells were color coded as red, green or no-color.

Single-cell RNA-sequencing

Four- (n=9) and 8-cell (n=4) embryos imaged and having two or more cells expressing at least one of the fluorescent proteins (RFP or GFP) were subjected to single-cell RNA-sequencing. Embryos were briefly treated with Tyrode's acid solution (Sigma) for removal of zona pelucida. The embryos were washed in PBS and incubated in Trypsin solution (Invitrogen) with RNase inhibitor (1IU/µl, Clontech) and BSA (50µg/µl) for the separation of the blastomeres. The blastomeres were separated by gentle pipetting, washed in PBS supplemented with RNase inhibitor and BSA, and snap frozen in individual micro-centrifuge tubes.

We amplified the cDNA of individual blastomeres according to the SMART-seq2 protocol (Picelli et al., 2014), followed by library preparation using Nextera XT DNA Sample Prep Kit (Illumina). The yields of cDNA and library amplification were assessed by Qubit (Invitrogen), and the quality of the distribution was assessed by Bioanalyzer (Agilent). Libraries were sequenced to produce pair-end reads of 100 nucleotides each in a HiSeq 2500 sequencer (Illumina).

Alignment of sequencing reads to fluorescent protein genes in brainbow construct v2.1

Sequencing reads from each cell were aligned to DNA sequences of the four fluorescent protein genes present on brainbow construct v2.1 (Addgene plasmid repository 18723) using STAR aligner. Reads that aligned exclusively to one of the sequences were used for counting. Cells were assigned a color coding (RFP, YFP, CFP or GFP) according to the greatest number of reads matching to one of the fluorescent proteins, or NA when no read aligned to any of the DNA sequences.

Alignment to the reference genome

STAR (STAR_2.5.1b, default parameters) was used to align single-cell sequencing reads to the reference genome (mm10, UCSC). Only uniquely aligned reads were used for the following analyses. Annotation files for Refseq genes and RepeatMasker defined repeats were downloaded from UCSC (February 2015), which contained a total of 24,225 genes and 5,138,231 repeats, respectively. These annotation files and the uniquely mapped reads in each cell were given as input to HTSeq-count (version 0.9.1) for counting of reads associated with each gene or repeat.

Principle component analysis

Principle component analysis was carried out based on all the genes that exhibited FPKM>1 in at least one blastomere.

Analysis of variance (ANOVA) analysis

A generalized linear model was fit to every gene as follows:

$$Y_{ijk}^{g} = u^{g} + \alpha_{i}^{g} + \beta_{ij}^{g} + \varepsilon_{ijk}^{g}$$

where Y_{ijk}^{g} is the observed expression level ($\log_2$(FPKM+1)) of gene g in embryo i, lineage j ($j = 0,1$), cell k; u^{g} is the average expression level of gene g in all cells; α_{i}^{g} is the embryo-effect to average expression; β_{ij}^{g} is the difference of expression levels between the two cell division lineages; ε_{ijk}^{g} is the error term. R package {stats} was used to implement this linear model and test for every gene the null hypothesis that no reproducible expression differences between the two division, namely $\beta_{ij}^{g} = 0$. To account for multiple hypothesis testing, R package {qvalue} was used to compute q-values from ANOVA derived p-values.

ANOVA analysis for TRENIs was carried out in the same way as for genes, except that index g in the above generalized linear model was used as the index for TRENIs.

Comparison of lineage variation and embryo variation

For every gene (g) we calculated the ratio (r^g) of lineage sum of squares to combined sum of squares of embryo and lineage as

$$r^g = SS^g_{lineage} \,/\, (SS^g_{embryo} + SS^g_{lineage}).$$

The random variable (R) of ratio of lineage sum of squares and combined sum of squares of embryo and lineage was defined as

$$R = \mathrm{SS}_{lineage}/(\mathrm{SS}_{embryo} + \mathrm{SS}_{lineage}).$$

Identification of novel transcript isoforms

Novel transcript isoforms were identified by Cufflinks (v2.2.1 with -g parameter) (Trapnell et al., 2012) with all Rainbow-seq data. Repeat sequences that overlapped with any Refseq annotated exons were removed from further analysis. TRENIs were identified by intersecting the remaining repeats with novel transcript isoforms using bedtools (2.27.0) (Quinlan and Hall, 2010).

Calculation of junction ratio

For each repeat sequence (indexed by i), HTSeq-count (version 0.9.1) was used to count the number of reads mapped to this repeat (T_i) and the number of reads spanning the junction of this repeat and nearby exons (S_i). Junction ratio (JR) for the ith repeat was calculated as $JR_i = T_i/S_i$.

Analysis of association between repeat families and TRENIs

Repeat families and the correspondence of individual repeat sequences to repeat families were retrieved from RepeatMasker (February 2015). A contingency table was built for each repeat family and TRENIs as follows.

	**Contained within TRENIs**	**Not contained in TRENIs**
**Is a member of this family**	a	b
**Not a member**	c	d

In the above table a, b, c, d are counts of repeat sequences and they sum to the total number repeat sequences (5,138,231). Odds ratio representing the degree of association between this repeat family and TRENIs was calculated as

$$OR = (a \times d)/(b \times c).$$

Ninety five percent confidence interval for odds ratio was calculated as

$$e^{\log(OR) \pm [1.96 \times SE(\log(OR))]},$$

where $SE(\log(OR)) = \sqrt{\frac{1}{a}+\frac{1}{b}+\frac{1}{c}+\frac{1}{d}}$.

Data and software availability

We deposited all sequencing data into GEO (GSE106287) for public access.

Reference

PICELLI, S., FARIDANI, O. R., BJORKLUND, A. K., WINBERG, G., SAGASSER, S. & SANDBERG, R. 2014. Full-length RNA-seq from single cells using Smart-seq2. *Nat Protoc,* 9**,** 171-81.

QUINLAN, A. R. & HALL, I. M. 2010. BEDTools: a flexible suite of utilities for comparing genomic features. *Bioinformatics,* 26**,** 841-2.

SCHNEIDER, C. A., RASBAND, W. S. & ELICEIRI, K. W. 2012. NIH Image to ImageJ: 25 years of image analysis. *Nat Methods,* 9**,** 671-5.

TRAPNELL, C., ROBERTS, A., GOFF, L., PERTEA, G., KIM, D., KELLEY, D. R., PIMENTEL, H., SALZBERG, S. L., RINN, J. L. & PACHTER, L. 2012. Differential gene and transcript expression analysis of RNA-seq experiments with TopHat and Cufflinks. *Nat Protoc,* 7**,** 562-78.

SUPPLEMENTARY FIGURES

Figure S1. Tamoxifen induced nuclear localization of Cre recombinase, related to Figure 2. (A) Overview of time and events. Tamoxifen is applied in a 2-hour window (yellow bar) at the early 2-cell stage. Microscopic analyses are carried out prior to (*), immediately after (**) and 1 hour after tamoxifen treatment (***). (B) Representative embryos (rows). Nuclear concentration of CRE was induced at the end of tamoxifen treatment (2nd, 3rd rows, two different embryos) and decreased to background level within 1 hour after removing tamoxifen (4th row).

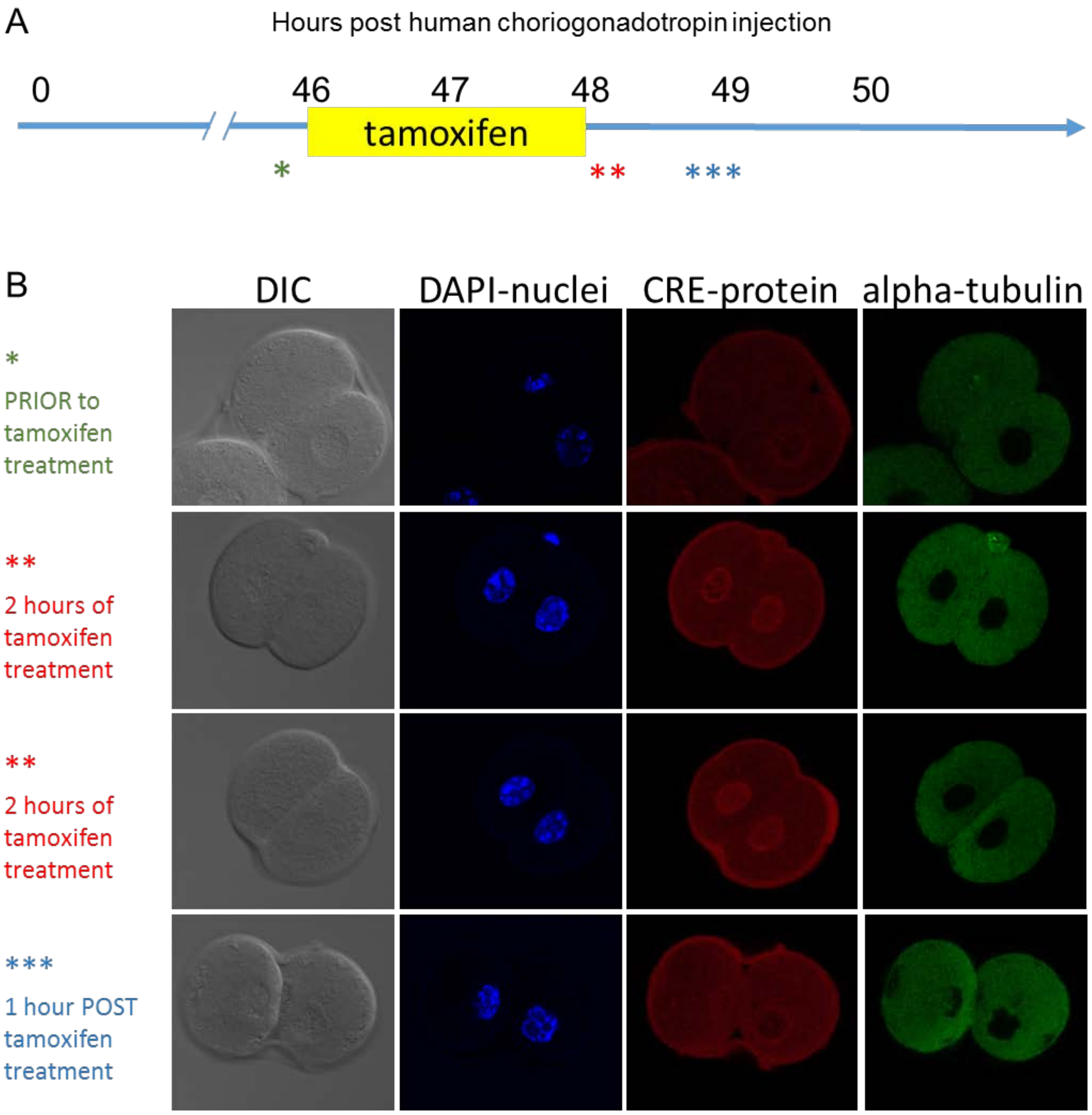

Figure S2. Overview of the single-cell sequencing data, related to Figure 3 and Figure 4. (A) Number of uniquely mapped read pairs (y axis) from each single cell (column). Each single cell is marked with an embryo number (Embryo), a cell number (Cell) and a binary lineage indicator (A or B). N: unresolved. (B) Principle component analysis of lineage resolved blastomeres at 4- (red) and 8-cell stages (green), using all genes with FPKM>1 in a least one blastomere.

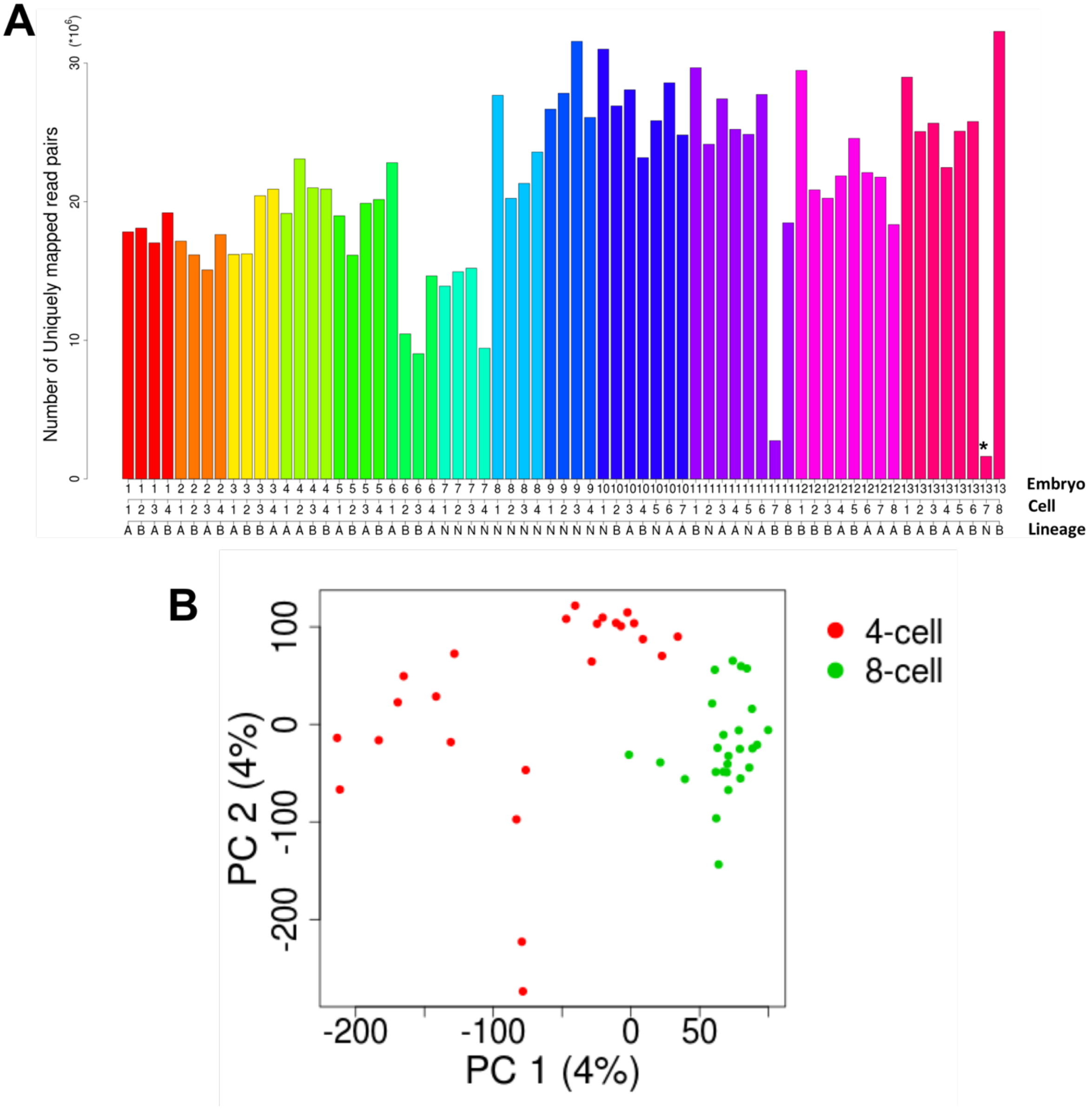

Figure S3. Histogram of TRENIs’ q-values derived from tests of equivalent expression between lineages at 4- (A) and 8-cell (B) stages, based from real data (red) and shuffled data (blue), related to Figure 5 and Figure 6. Lower panels: expansion of the range 0 – 0.4 showing difference of real data and shuffled data in the range of small q-values.

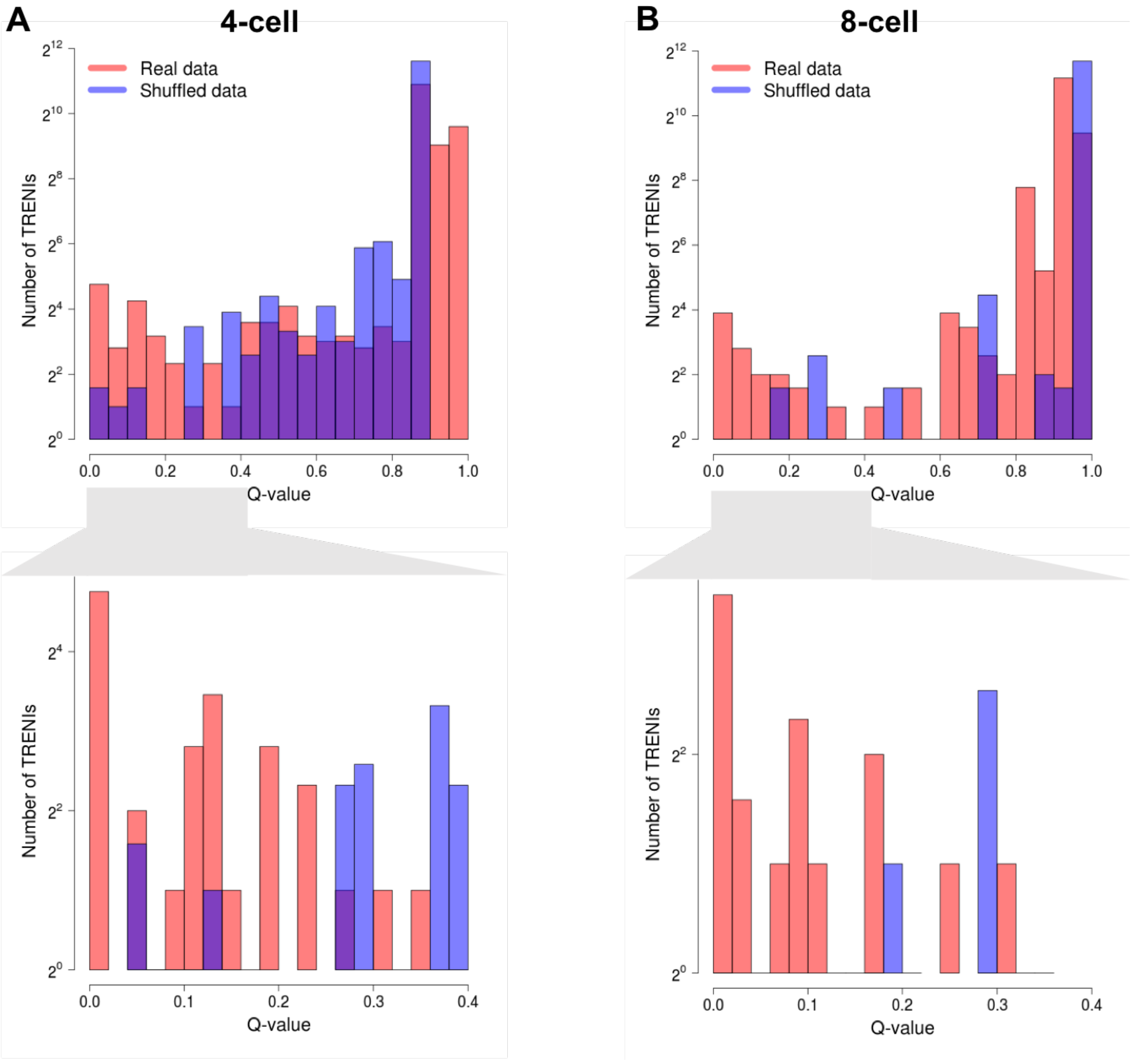

Figure S4. Tests of equivalent expression between lineages for all expressed repeat sequences, related to Figure 5. Repeats' q-values derived from real (red) and shuffled data (blue) exhibited similar histograms, in both 4- (A) and 8-cell (B) stages.

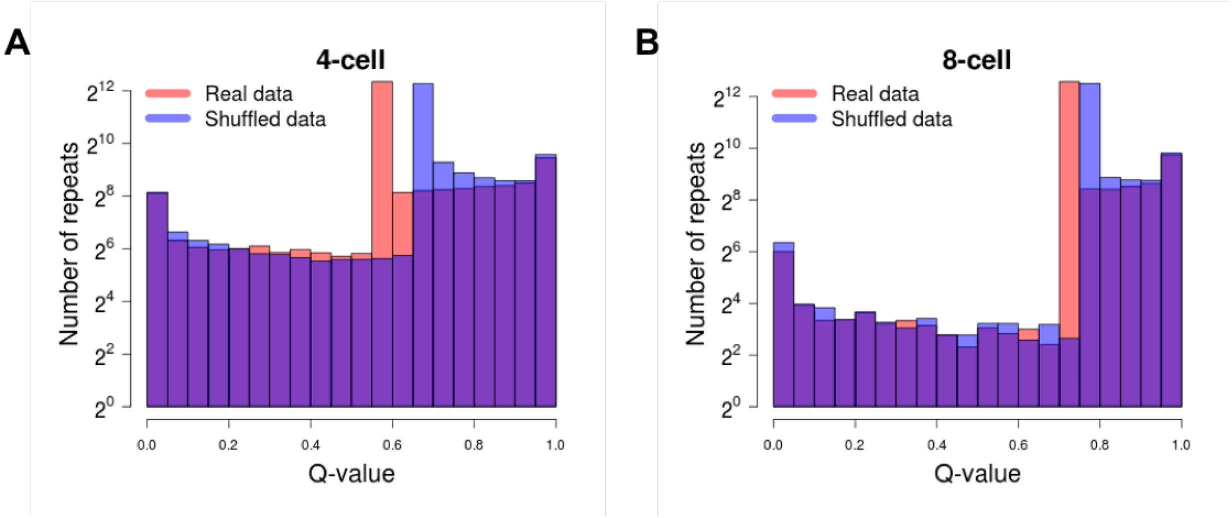

Figure S5. Sensitivity analysis of transposon related novel isoforms (TRENIs), related to Figure 5. Odds ratios between TRENIs and each transposon family (columns), calculated from only the repeat sequences with junction ratio (JR) > 0.9 (blue) were not very different from those calculated from all TRENI embedded repeats (red). Odds ratio was not calculated when the TRENIs of a repeat family were fewer than 20. Vertical bars: 95% confidence intervals.

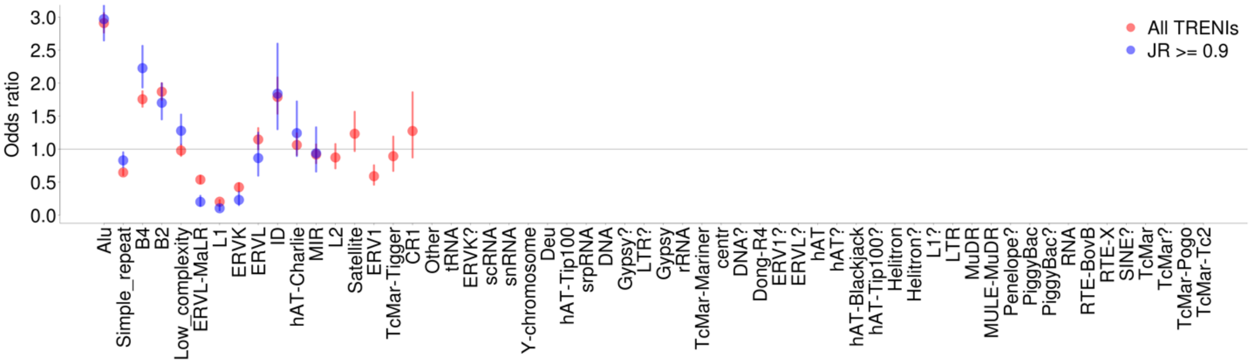

Figure S6. Novel transcript isoforms of *Gata4* and *Khdc1a* containing murine Alu in 5' UTRs, related to Figure 5 and Figure 6. (A) Expression levels of a novel transcript isoform of *Gata4* in 4-cell blastomeres. (B) A novel transcript isoform of *Khdc1a* in 8-cell blastomeres. FPKM (y axis) of every blastomere (column) in each embryo (marked by embryo number in columns). Shaded columns delineate different embryos. The blastomeres of the two lineages are marked with A (red) and B (blue), respectively.

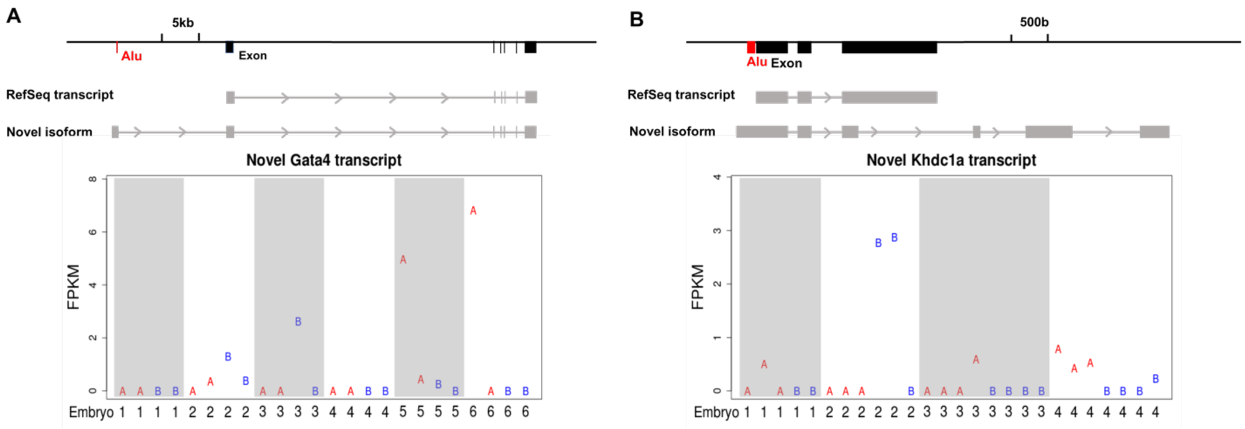

SUPPLEMENTARY TABLES

Table S1. Summary of Rainbow-seq data from 4-cell stage blastomeres, related to Figure 1, Figure 3, and Figure 4. Each blastomere (row) is indexed by embryonic stage (Stage), embryo number (Embryo), cell number (Cell), cell division lineage (Lineage), the total number of Rainbow-seq reads (# of reads), number (# uniquely mapped) and the percentage (Genome mapping rate) of reads uniquely mapped to the mm10 genome, and the percentage of reads mapped to the mitochondria genome (Mitochondria mapping rate). NA: undetermined.

Stage	Embryo	Cell	Lineage	# of reads	# uniquely mapped	Genome mapping rate	Mitochondria mapping rate
4-cell	E1	B1	A	21314126	17819219	83.60%	3.66%
		B2	B	21985294	18088361	82.27%	4.98%
		B3	A	20235470	17028354	84.15%	1.47%
		B4	B	23513067	19197409	81.65%	6.23%
	E2	B1	A	20704467	17146739	82.82%	5.54%
		B2	B	20058467	16160003	80.56%	7.39%
		B3	A	18278090	15080241	82.50%	5.29%
		B4	B	21622583	17630799	81.54%	7.12%
	E3	B1	A	18775617	16185096	86.20%	2.57%
		B2	B	18793826	16228376	86.35%	3.96%
		B3	B	23865180	20424658	85.58%	3.30%
		B4	A	24696310	20903704	84.64%	4.72%
	E4	B1	A	25954803	19148778	73.78%	5.02%
		B2	A	33404660	23079578	69.09%	6.49%
		B3	B	29530032	21001228	71.12%	7.07%
		B4	B	31340190	20908747	66.72%	7.94%
	E5	B1	A	26738657	18974243	70.96%	5.28%
		B2	B	28170918	16139013	57.29%	30.15%
		B3	A	28801088	19877650	69.02%	4.79%
		B4	B	28915622	20154724	69.70%	21.79%
	E6	B1	A	29629536	22814400	77.00%	3.00%
		B2	B	12599757	10457443	83.00%	6.21%
		B3	B	10829517	9033260	83.41%	2.13%
		B4	A	17688808	14640436	82.77%	1.81%
	E7	B1	NA	16610220	13908974	83.74%	12.83%
		B2	NA	17463191	14944165	85.58%	5.43%
		B3	NA	18099348	15200717	83.98%	7.91%
		B4	NA	11109883	9425697	84.84%	6.59%
	E8	B1	NA	32556574	27666944	84.98%	3.47%
		B2	NA	23739102	20243893	85.28%	2.85%
		B3	NA	25140202	21316100	84.79%	3.13%
		B4	NA	28565034	23582751	82.56%	2.94%
	E9	B1	NA	32027811	26666074	83.26%	8.36%
		B2	NA	36935316	27818485	75.32%	7.19%
		B3	NA	44737967	31564011	70.55%	8.46%
		B4	NA	35022576	26073115	74.45%	13.18%

Table S2. Summary of Rainbow-seq data from 4-cell stage blastomeres, related to Figure 1, Figure 3, and Figure 4. Each blastomere (row) is indexed by embryonic stage (Stage), embryo number (Embryo), cell number (Cell), cell division lineage (Lineage), the total number of Rainbow-seq reads (# of reads), number (# uniquely mapped) and the percentage (Genome mapping rate) of reads uniquely mapped to the mm10 genome, and the percentage of reads mapped to the mitochondria genome (Mitochondria mapping rate). NA: undetermined.

Stage	Embryo	Cell	Lineage	# of reads	# uniquely mapped	Genome mapping rate	Mitochondria mapping rate
8-cell	E10	B1	NA	35510447	30994895	87.28%	2.55%
		B2	B	31310655	26908056	85.94%	4.20%
		B3	A	32364998	28069936	86.73%	3.32%
		B4	B	26879062	23168806	86.20%	3.03%
		B5	NA	30026238	25835885	86.04%	2.17%
		B6	A	33728624	28568517	84.70%	2.24%
		B7	A	29108703	24806968	85.22%	8.06%
	E11	B1	B	38092133	29663146	77.87%	5.68%
		B2	NA	30719208	24136791	78.57%	4.52%
		B3	A	35052929	27408245	78.19%	7.15%
		B4	A	34218985	25218809	73.70%	7.74%
		B5	NA	32137812	24846522	77.31%	7.91%
		B6	A	33420557	27727130	82.96%	7.07%
		B7	B	3650871	2758113	75.55%	11.98%
		B8	B	22651287	18476451	81.57%	10.44%
	E12	B1	B	34015604	29465623	86.62%	3.54%
		B2	B	24573413	20847753	84.84%	4.69%
		B3	B	23764288	20252119	85.22%	4.72%
		B4	A	26254436	21857322	83.25%	4.68%
		B5	B	29526449	24568457	83.21%	4.23%
		B6	A	26966760	22102352	81.96%	3.96%
		B7	A	28616287	21771577	76.08%	5.21%
		B8	A	22578208	18345514	81.25%	4.97%
	E13	B1	B	34150397	28982368	84.87%	3.90%
		B2	A	29319315	25052456	85.45%	3.33%
		B3	B	31016258	25656830	82.72%	4.22%
		B4	A	26313847	22461091	85.36%	3.46%
		B5	A	29808796	25077571	84.13%	2.66%
		B6	B	31502252	25769136	81.80%	3.55%
		B7	NA	2967324	1618930	54.56%	0.01%
		B8	B	41717993	32283499	77.39%	5.65%
